# Implementing a video-based intervention to empower staff members in an autism care organization: a qualitative study

**DOI:** 10.1186/s12913-016-1820-9

**Published:** 2016-10-21

**Authors:** Alex Hall, Tracy Finch, Niina Kolehmainen, Deborah James

**Affiliations:** 1 Research and Enterprise Services, Newcastle University, Newcastle upon Tyne, NE1 7RU UK; 2 Present address: School of Health Sciences, University of Manchester, Oxford Road, Manchester, M13 9PL UK; 3 Institute of Health and Society, Newcastle University, Baddiley-Clark Building, Richardson Road, Newcastle upon Tyne, NE2 4AX UK; 4 Faculty of Health and Life Sciences, Northumbria University, Coach Lane Campus East, Newcastle upon Tyne, NE7 7XA UK

**Keywords:** Complex interventions, Video feedback, Empowerment, Autism, Qualitative, Implementation, Normalization process theory

## Abstract

**Background:**

Implementing good-quality health and social care requires empowerment of staff members within organizations delivering care. Video Interaction Guidance (VIG) is an intervention using positive video feedback to empower staff through reflection on practice. This qualitative study explored the implementation of VIG within an autism care organization in England, from the perspective of staff members undergoing training to deliver VIG.

**Methods:**

Semi-structured interviews were conducted with a purposive sample of 7 participants working within the organization (5 staff undergoing training to deliver VIG; 2 senior managers influencing co-ordination of training). Participants were asked about their views of VIG and its implementation. The topic guide was informed by Normalization Process Theory (NPT). Data were analysed inductively and emerging issues were related to NPT.

**Results:**

Five broad themes were identified: (1) participants reported that they and other staff did not understand VIG until they became involved, initially believing it would highlight negative rather than positive practice; (2) enthusiastic feedback from staff who had been involved seemed to encourage other staff to become involved; (3) key implementation challenges included demands of daily work and securing managers’ support; (4) ideas for future practice arising from empowerment through VIG seemed difficult to realise within an organizational culture reportedly unreceptive to creative ideas from staff; (5) individuals’ emotional responses to implementation seemed beyond the reach of NPT, which focused more upon collective processes.

**Conclusions:**

Implementation of VIG may require recognition that it is not a ‘quick fix’. Peer advocacy may be a fruitful implementation strategy. Senior managers may need to experience VIG to develop their understanding so that they can provide appropriate implementation support. NPT may lack specificity to explain how individual agency weaves with collective processes and social systems to embed innovation in routine practice. This exploratory study has provided broad insights into facilitators and barriers to the implementation of an intervention to empower staff within an autism care organization. Further research is needed into similar interventions, including a focus upon staff members’ emotional responses and resources, and how such interventions may relate to the culture of the organization in which implementation occurs.

**Electronic supplementary material:**

The online version of this article (doi:10.1186/s12913-016-1820-9) contains supplementary material, which is available to authorized users.

## Background

It is increasingly acknowledged that implementing good-quality health and social care requires the empowerment of staff members within organizations delivering care [1–3]. Since the 1940s, organizational research has recognised that initiatives to empower staff members enhance their job performance, positive attitudes to work, and emotional well-being [4]. In a scoping review of person-centred planning in social care, Dowling et al. [5] stated that one way in which good care organizations empower their staff is by providing training and support according to the particular needs of staff, to allow them to reach their maximum potential as individuals. Empowerment may be understood as a process by which people ‘gain mastery over their affairs’ [6], and has close links with advocacy as a mechanism for helping people speak up for themselves and influence decisions [7].

Commitment to an organizational culture in which staff are empowered, valued for their skills and potential, and encouraged to speak for themselves and influence decisions, can be difficult to sustain in organizations perennially engaged in short-term firefighting of one crisis after another [8]. This difficulty may be particularly pronounced in settings such as intellectual disability and autism care in which staff often face high levels of behaviour that challenges from service users [9]. Structure and routine can help reduce the frequency of such behaviour, but can lead to staff feeling disempowered by a task-orientated approach to work which does not afford them scope to influence practice and develop relationships with service users [10]. Staff faced with high levels of behaviour that challenges often experience of a range of negative emotions, and have to rely upon emotional detachment (thinking of their role as ‘just a job’ with no personal importance) as a coping strategy to avoid burnout [9]. Establishing an organizational culture in which staff feel empowered is therefore likely to require organizations to pay attention to the well-being of their staff by recognising the emotional demands of caring roles and providing support networks to help staff cope with these demands [5].

This present study is concerned with an intervention with potential to empower staff, called Video Interaction Guidance (VIG) [11]. VIG is a complex intervention [12] based around positive video feedback of successful interactions to help staff appreciate what they are doing well and empower them to make changes to their practice. VIG is underpinned by a constructivist, critical pedagogy which advocates a collaborative approach valuing staff as active agents in the learning process, rather than a didactic approach which positions staff as passive agents awaiting instruction [13]. VIG and similar interventions employing positive video feedback have been found to be effective for empowering staff in range of health, social care and educational contexts by enhancing their interactional skills through heightened attention to verbal, non-verbal, and paralinguistic components of communication [14]. VIG and similar interventions have also been endorsed by National Institute for Health and Clinical Excellence guidelines focusing upon developing relationships between caregivers and young children [15] and children and young people with autism [16]. Despite a growing evidence-base and endorsement of VIG, understanding how to implement VIG as an intervention to support staff within organizational contexts is lacking. To our knowledge, to date only one, non-peer-reviewed paper [17] has explored this area, but that work focused upon using VIG to enhance interactions between staff as part of a peer support strategy, rather than interactions between staff and service users.

It is increasingly accepted that implementing and sustaining interventions within health and social care is extremely challenging, influenced by a diverse range of factors such as features of the intervention, individual-level attitudes towards the intervention, and organizational-level factors and context [18]. Implementation science approaches are likely to have an important role in understanding the implementation of interventions because of their consideration of theoretical frameworks which may help to predict and explain successful change [19]. In this present study we applied Normalization Process Theory [NPT], a mid-range sociological theory which seeks to account for how novel interventions become routine components of everyday practice [20, 21]. NPT consists of four main constructs to understand the practical work that is involved in making an intervention work: *Coherence* refers to how people make sense of the intervention (e.g. how they differentiate it from existing practice); *Cognitive Participation* refers to people’s involvement with the intervention (e.g. the influence of key people driving implementation); *Collective Action* refers to work done in enacting the intervention (e.g. the mix of skills that people have between them); *Reflexive Monitoring* refers to the work done in evaluating and appraising the intervention (e.g. the perceived impact of the intervention, and its visiblility to people) [21]. These four constructs are not linear components; rather, they exist in reciprocal, generative relationships with one another and within the specific context in which the intervention is employed [21, 22]. NPT was chosen because it addresses both individual and organizational factors involved in the embedding of interventions into routine practice, and explicitly focuses on the interactions between these factors as people work together collaboratively in professional settings [21, 22]. It is a mid-range theory that can be applied to focus on local contexts and variations rather than a grand theory operating at a universal level [23]. Mid-range theories are designed to help researchers navigate empirical enquiry [24]. A recent systematic review of the use of NPT to research implementation processes [25] found that many researchers reported that they benefitted from applying NPT as an heuristic conceptual framework and from thinking critically about its relevance, rather than being constrained by a rigid adherence to its constructs.

The aim of the present study was to explore the implementation of VIG within an autism care organization, using NPT as a theoretical lens to understand facilitators and barriers to successful implementation.

## Methods

### Study design and context

The study was a qualitative exploratory inquiry using semi-structured interviews with staff working in an autism care organization, ‘Organization A’. It was located within a project which aimed to help empower staff members of Organization A through the use of VIG. Organization A is a registered charity in the North of England established over 30 years ago providing educational, residential and community services for children, young people and young adults with autism. At the time of the project, Organization A’s educational facilities included two schools, and a post-16 college split across two campuses. Residential facilities included properties located within residential communities; typically each property was home to four service users with a dedicated team of staff providing 24-h care. Community services offered a range of social and leisure outreach activities and short respite breaks. Organization A employed around 450 staff members in a range of professional and support roles, including teachers, classroom support workers, residential support workers, speech and language therapists, an occupational therapist, and community activity workers.

There were two aspects to the implementation of VIG. The first aspect was to deliver VIG to individual staff members of Organization A to help them reflect upon their interactions with service users. Evaluative work regarding the impact of VIG from the perspective of staff receiving it has been published elsewhere [26, 27]. Key findings were that receiving VIG seemed to be a positive experience for staff; it had afforded them the time to step out of the intensity of daily work and allowed them to reflect on their practice, and as a result they experienced benefits such as improved self-confidence in their own skills, an increased conviction to suggest positive changes to practice based on a deeper understanding of service users, and a desire to seek more diversity in their work roles [26, 27]. The second aspect was to train a small number of staff members to be able to deliver the intervention themselves. The rationale for this aspect was that by training some staff to be able to deliver the intervention, capacity for using the intervention would be left behind in Organization A after the end of the project without the need for continual external input, thus helping to make the intervention sustainable for future use. This present study explores the implementation of VIG from the perspectives of staff involved in training to deliver VIG. The intervention is described in Table 1.

**Table 1:** Video interaction guidance - the intervention

VIG is delivered by a trained facilitator (hereafter referred to as a ‘Guider’) working with an individual staff member. Delivering the intervention requires non-specialist video technology such as a simple video camera available from any high street retailer, and basic video editing software available as standard on most PCs. There are five main steps undertaken in the delivery of VIG:	
Step 1	The Guider and staff member *negotiate the goals* that the staff member wishes to work towards (e.g. a support worker might wish to understand how better to help a service user to communicate with them more in classroom activities).
Step 2	The Guider *films a brief video* (10–20 min) of the staff member engaged in typical interaction with the service user (e.g. the support worker and the service user engaged in a classroom activity together).
Step 3	The Guider *reviews and edits the video* to extract moments of successful interaction between the staff member and service user. This step involves application of a set of interactional behavioural principles founded upon the psychological concept of intersubjectivity (how people are innately receptive to subjective states in others), which at their most basic include behaviours such as shared eye gaze and mirroring of posture [44, 45].
Step 4	The Guider *feeds back* the edited moments to the staff member, with emphasis placed on giving the staff member space to reflect upon what they are seeing in relation to their own emotions, beliefs, and contextual knowledge. This discursive style is grounded in established constructivist pedagogical approaches of Zone of Proximal Development [46] and Scaffolding [47].
Step 5	The Guider and staff member *repeat steps 1–4* until the staff member feels satisfied that they have achieved their goal. Usually three to four repetitions are sufficient.

### VIG Guider training

Training a small number of staff to become VIG Guiders (hereafter referred to as ‘trainee Guiders’) began with a two-day induction course delivered by qualified VIG Guiders, in which trainee Guiders were introduced to the principles and theoretical underpinnings of VIG. Following their two-day induction course, trainee Guiders worked under the supervision of qualified Guiders through three phases of training, each taking around six months to complete. Phase one required a trainee Guider to recruit a minimum of two staff members who were interested in receiving VIG to develop their practice. Phase one required the trainee Guider to work through six ‘cycles’ (i.e. six iterations of the video and feedback process outlined in steps one to four of the intervention description provided above) with these staff (six in total rather than six per staff member), and engage in seven one-hour supervision sessions. In phase one, trainee Guiders mainly developed technical skills in taking video, identifying moments of successful interaction, video editing, and beginning to discuss the video clips with staff. Phase two required a trainee Guider to work through nine further cycles with an additional three staff members, engaging in eight supervision sessions. Phase three required a trainee to work through 12 further cycles with an additional three staff members, engaging in eight supervision sessions. By phases two and three the technical skills of taking and editing video are usually well-established and these later phases focus further upon the development of the negotiation of goals, and the discursive feedback style described earlier. In the same model of working as the intervention itself, trainee Guiders videoed the feedback sessions they held with the staff members and discussed clips from these feedback sessions with their supervisor in order to reflect upon their own skills as trainee Guiders. Therefore, the model of collaborative discussions around positive moments of interaction captured on video was replicated in both the delivery of intervention (i.e. in feedback sessions between trainee Guider and staff member, in which the video focus was the staff member’s interaction with a service user) and the Guider training (i.e. in supervision sessions between supervisor and trainee Guider, in which the video focus was the trainee Guider’s interaction with the staff member during the feedback session). Progression from one phase to the next required trainee Guiders to submit examples of their feedback sessions with staff for discussion and accreditation by a qualified VIG Guider who was not involved with the project. For further information about VIG Guider training, see AVIGuk [28].

Training VIG Guiders was implemented in a cascade model over the duration of the project, designed to allow trainees Guiders to reach Guider level and subsequently train other staff of Organization A to become Guiders. The cascade model also meant that the number of staff who were directly receiving VIG would increase exponentially with an increasing number of trainee Guiders.

At the beginning of the project, the fourth author (DJ) supervised the training of the first author (AH) and one staff member of Organization A to become VIG Guiders. Later in the project, these three people provided supervision to additional trainee Guiders. The first (AH) and fourth authors (DJ) were consulted by senior management of Organization A regarding the suitability of staff considered for VIG Guider training, but the ultimate identification of trainee Guiders was made by senior management.

### Participants and recruitment

Participants were eligible for inclusion if they were employed by Organization A and (i) a trainee Guider OR (ii) had been trainee Guiders but had withdrawn from training OR (iii) a senior manager with influence over the co-ordination of Guider training. These criteria resulted in 25 eligible participants. Two were deemed ineligible as they were being trained by the first author (AH) who would be responsible for data collection; it was felt that this closeness of relationship might adversely affect trustworthiness of the data. The final number of participants eligible for inclusion was 23. Eight were purposively sampled to provide maximum variation [29] in the service areas of the organization, working patterns, levels of involvement in training, and the points during the project at which they commenced training. Seven of the sampled participants were available to participate. These seven were all full-time employees. Three were currently involved in Guider training (a speech and language therapist, a college tutor and an adult home deputy manager), two had been trainee Guiders but had withdrawn (a teacher and a children’s home manager), and two were senior managers. Five of these participants were female and two were male. The eighth sampled participant was a part-time female employee in the community services area but had left the organization by the time the study was conducted. There were no other eligible participants from the community services area and hence no further participants were sampled. Although not directly training any of the sampled participants, the first author (AH) had developed working relationships with each sampled participant over the course of the project and rapport was established.

### Ethics, consent and permissions

Participants were contacted by the first author (AH) via email and provided with written and verbal information about the study. This information stated that anything participants disclosed regarding named individuals would be treated anonymously except if any safeguarding issues became apparent, in which case a senior manager would be made aware. Written consent was sought from participants prior to data collection. Ethics approval was granted by Newcastle University Research Ethics Committee as an amendment to the ethics application for the main project in which this present study was situated (no. 7327).

### Materials and procedures

The first author (AH) developed an interview topic guide loosely based upon the four constructs of NPT. NPT provides detail of different mechanisms within the four constructs, however the interview questions were based as open questions at the most general level of the constructs, thus allowing participants scope to raise issues important to them within the broad constructs. The topic guide is available as an Additional file 1; examples include:

- *“What was your understanding of VIG when you were introduced to it?”* was based on the construct of *Coherence*, which attempts to establish how participants made sense of the intervention;
- *“whose involvement do you see as necessary for VIG to have maximum impact?”* was based on the construct of *Cognitive Participation*, which explores specific roles within implementation;
- *“Can you tell me how a piece of VIG work gets done?”* was based on the construct of *Collective Action*, which explores participants’ views about the practical work of carrying out the intervention;
- *“Do you think VIG has had any impact within the organisation?”* was based on the construct of *Reflexive Monitoring*, which explores evaluation and appraisal of the intervention.

The topic guide was first piloted by the first author on himself, since he had completed VIG Guider training during the main project. The topic guide was then refined and further piloted with a trainee Guider who was not included in the study. Interviews were conducted in a manner which followed the participant, rather than forcing the participant to follow specific questions based on NPT constructs in a linear fashion. Interviews took place in a quiet location of each participant’s choosing (e.g. an office in their workplace). Each interview lasted until participants indicated they did not wish to elaborate any further (around 30 min) and was audio recorded. Interviews were conducted during the final few months of the project.

### Analysis

A two-stage analytical approach was adopted. The first stage was informed by the first two phases of inductive thematic analysis [30]. The first author (AH) familiarised himself with the data by transcribing each interview verbatim, reading and re-reading the transcripts, and noting initial impressions. The first author (AH) then generated a set of data-driven codes from the transcripts, discussed the coded data with second author (TF), and further developed the codes. The second stage involved comparing the codes with the constructs of NPT [31], and exploring how they related to one another. This use of NPT was to allow us to better understand the processes and interactions, rather than the discrete things that participants say which emerge from an entirely inductive initial analysis. This second stage involved establishing the meaning of each NPT construct in relation to the intervention, by considering the individuals involved, the work they do, the specifics of VIG, and the overall organizational context of Organization A [31]. The first author (AH) worked in an iterative process moving back and forth between the meaning of each code and the constructs of NPT to identify fit and non-fit between the codes and the NPT constructs, and continually discussed the relationship between the codes and the NPT constructs with the second author (TF). The third (NK) and fourth authors (DJ) provided critical analysis and interpretation of the results.

### Quality assurance

Credibility, transferability, dependability and confirmability have been proposed as four tenets of trustworthiness in qualitative research [32] (Additional file 2).

- Credibility: the first (AH) and fourth authors (DJ) had intimate knowledge of the organizational context, maintaining prolonged engagement with the implementation of VIG within Organization A throughout the project. Data disclosed by participants to the first author (AH) reflected established rapport as they included personal views (see Results section). Amongst the authors there is an understanding of the wider context of health and social care services: the first author (AH) has previous experience as a support worker with people with autism and intellectual disabilities; in addition to being experienced health services researchers, the third author (NK) is a practising occupational therapy clinician and the fourth author (DJ) is a registered speech and language therapy clinician.
- Transferability: detailed description of Organization A; reporting of sampling frame and inclusion criteria.
- Dependability & confirmability: The first author (AH) kept an audit trail of data analysis. The second (TF) and third authors (NK) were not involved in the main project. The second author (TF) is a core member of the academic team responsible for NPT. In order to guard against influence arising from the ‘closeness’ of the first author (AH) to the participants, and from the second author (TF) to NPT, the third (NK) and fourth authors (DJ) provided critical comment on the results. We also paid attention to data that was less closely aligned to the constructs of the NPT framework to guard against ‘forcing’ data into NPT.

## Results

Seven participants were interviewed. Inductive coding of interview transcripts generated a range of codes; those that were finally agreed to as the ultimate key codes are presented in Table 2. From further relating these codes to the NPT constructs (Table 3), five higher level themes emerged: (1) *First steps: What is it all about?*; (2) *Will it be worth it? Perceived potential of VIG*; (3) *Doing VIG work*; (4) *Ideas for the future: New directions or same path?*; (5) *What about the individual? The role of emotions within implementation*. The results are presented below, according to these themes. Each section presents the key inductive results, before developing the inductive analysis by illustrating the relationships with the NPT constructs, and the relationships between the NPT constructs.

**Table 2:** Final codes generated during inductive analysis

Code	Definition
Expectations	Expectations of VIG e.g. whether they felt it would succeed, prior to getting involved
Feedback from staff receiving VIG	Feedback from a staff member who has received VIG
Getting involved in VIG – personal involvement	How trainee Guiders and managers came to be involved with VIG
Getting involved in VIG – staff receiving VIG	How staff receiving VIG became involved
Ideas for use of VIG	How VIG might work within the organization in future - ideas which are not realized yet but might happen
Impact of daily role	How the trainee Guider’s daily role related to their ability to do VIG work
Meanings of VIG	Perception and understanding of VIG within the organization
Own role in relation to VIG	How participants see themselves as agents in the continuation of VIG
Perception and evaluation of the work	What was easy or difficult about VIG work
Priorities of service, unavoidable problems	Organizational priorities which might impact upon VIG work
Reach of VIG	Perception of the organizational scope of VIG – how wide do participants think the VIG ‘net’ might be cast?
Reactions to VIG	Reactions at time of introduction to VIG, and how they developed over time, including after having experienced VIG
Reflective practice	How reflective practice is undertaken within the organization
Relationships between those involved in VIG	Relationships with, or knowing staff involved in VIG; relationships with managers; relationships with service users
Resources to help VIG	Resources which they think would be necessary to ensure continuation of VIG
Role of VIG Guider	Any mention of the VIG Guider specifically
Seeing, showing, watching, visual image	Any references to the visual aspect of images generated within VIG
Strategic co-ordination	References to role that management play in either supporting individual VIG Guiders, or supporting VIG within the organization
Talking about VIG	Sharing of VIG within the organization; the level of awareness about VIG within the organization
VIG terminology and references to training criteria	Intervention-specific jargon, including criteria of Guider training
What could have been improved	Suggestions of what could have been better

**Table 3:** Codes related to NPT constructs

Coherence	
Reactions to VIG	
Getting involved – personal involvement	
Getting involved – staff receiving VIG	
Own role in relation to VIG	
Talking about VIG	
Expectations	
Impact of daily role	
Priorities of service	
Meanings of VIG	
Seeing, showing, watching, visual image	
VIG terminology and training criteria references	
Ideas for use of VIG	
Cognitive Participation	
Reactions to VIG	
Getting involved – personal involvement	
Getting involved – staff receiving VIG	
Own role in relation to VIG	
Talking about VIG	
Resources to help VIG	
Ideas for use of VIG	
Collective Action	
Relationships between those involved in VIG	
Strategic co-ordination	
Impact of daily role	
Priorities of service	
Reach of VIG	
Role of VIG Guider	
Resources to help VIG	
Reflective practice	
VIG terminology and training criteria references	
Ideas for use of VIG	
Reflexive Monitoring	
Reactions to VIG	
Perception and evaluation of the work	
Talking about VIG	
Own role in relation to VIG	
What could be improved	
Ideas for use of VIG	

### First steps: what is it all about?

This theme explores participants’ own first impressions of VIG, and their reports of their colleagues’ first impressions. One trainee Guider reflected on the experience of a rather blunt introduction to Guider training and described how an initial understanding about the intervention may have helped ease her introduction:

*I got a phone call from my manager…saying I’d been put forward for VIG [Guider training]…I had to book my own place [on the induction course] and then I was given very little or no information until that day when I went up there… that was quite a horrendous experience… I was quite horrified because I felt like a fish out of water because I felt as if everybody knew what they were doing and I’m sitting there trying to catch up. And I’m quite a slow learner anyway, so I really, really, really felt out of my depth* (Trainee Guider 1, children’s home manager)

Trainee Guiders reported that both their own and staff members’ understanding of VIG was only fully realised once they had become involved with the intervention. The same trainee Guider stated how she had attempted to explain VIG to one of her staff members who was interested in receiving the intervention, but that he only understood properly once after he had started receiving the intervention:

*I could explain [VIG] til the cows come home to [name of colleague] but he didn’t quite understand it until he was actually involved* (Trainee Guider 1, children’s home manager)

Trainee Guiders reflected that the general understanding of VIG amongst staff, and considerations of whether or not to receive the intervention, appeared to be shaped by powerfully established beliefs about work that was usually conducted within the organization. VIG’s focus upon moments of successful practice as a starting point for staff development appeared to be rather different to the historical culture of the organization which could be perceived as a culture of explicitly addressing negative practice. As a result of this perception, there was strong scepticism at the beginning of the project about the presentation of VIG as an alternative way of working:

*people had a lot of apprehensions about it, it was kind of billed as this positive thing…but from knowing what had happened in the past… it sounded like [Organization A] was kind of putting a positive spin on it* (Trainee Guider 4, college tutor)

These findings appeared to relate to the NPT constructs of Coherence (understanding the intervention) and Cognitive Participation (involvement with the intervention). Participants’ descriptions of VIG suggested that the ways in which they and other staff members within Organization A understood the intervention seemed to work in reciprocity with their involvement in the intervention. Members of staff seemed to feel that they had some sense of understanding about VIG prior to becoming involved with it; either a lack of detailed understanding, or a particular understanding shaped by their perceptions of previous initiatives within Organization A. However, their sense of understanding appeared to develop and change as they became involved in working with VIG. This finding seemed to highlight a reciprocal, generative nature between the constructs of Coherence and Cognitive Participation; which is developed further in the following theme.

### Will it be worth it? Perceived potential of VIG

This theme develops the first theme by exploring how initial impressions about VIG may have been revised once the implementation of VIG had begun within the organization. Trainee Guiders reported that staff members’ assumptions that VIG would focus upon negative practice were contradicted after experiencing an increase in confidence as a result of receiving the intervention. Some of these staff then became enthusiastic advocates for the intervention:

*she [staff member who had received VIG] started with the same attitude, that people were going to be looking for negative things… but by the end she was so confident she was selling it to other people* (Trainee Guider 2, teacher)

Staff members advocating VIG to their colleagues seemed potentially a more fruitful implementation strategy for trainee Guiders to find staff members to work with, rather than relying upon staff to be instructed to receive VIG by a senior manager removed from the immediate staff team:

*when it comes from someone that you work with on a day to day basis, it’s a lot better than coming from someone kind of higher up saying “have you thought about this”… [better] if it comes from a friend rather than if it came from a manager* (Trainee Guider 4, college tutor)

One trainee Guider who had withdrawn from training was a line manager who seemed to desire a sense of continued involvement with the intervention by promoting VIG amongst her staff team:

*I think it’s brilliant, it’s just a shame I wasn’t part of it, but you know.. I can be as a manager by introducing staff to it* (Trainee Guider 1, children’s home manager)

This quote suggested that this trainee Guider’s own experience of VIG left a lasting positive impression upon her despite circumstances forcing her to withdraw from Guider training: she seemed to be looking to her role as a line manager to keep herself involved with the implementation of VIG within the organization by encouraging her staff team to receive the intervention because she felt that it was ‘brilliant’.

For some trainee Guiders, there was the sense of an ongoing battle of understanding where the delivery of VIG, and application of the skills learned in Guider training, fitted conceptually in relation to their daily role. For one trainee Guider, there was the sense that the intersubjective principles underpinning VIG feedback sessions, including allowing space in conversations for staff to offer opinions, were beginning to filter into his regular supervisory role with staff:

*I think it’s [Guider training] definitely helped in my wider role, without a doubt, when I’m doing supervisions and mentoring, I look at that a lot more from the other member of staff’s point of view than just trying to get my point across* (Trainee Guider 4, college tutor)

However another trainee Guider at the same phase of training seemed to have a very separate conception of VIG work and her daily role as a supervisor to staff, suggesting that understanding how VIG might complement daily practice could be challenging:

*I’ve never actually mentioned it [VIG] in the [regular] supervision. I don’t know why, I just haven’t* (Trainee Guider 6, adult home deputy manager)

This theme appeared to show how the relationship identified in the first theme between the NPT constructs of Coherence (understanding) and Cognitive Participation (involvement) was enhanced by the construct of Reflexive Monitoring (evaluation and appraisal). Positive evaluations from staff who had received VIG seemed to influence other staff members’ understanding of VIG and encourage them to become involved in receiving it themselves. Yet despite an apparently strong influence of positive evaluation upon understanding and involvement, it seemed that some of the trainee Guiders may have struggled to understand quite how they might assimilate VIG within their wider roles. This suggested that the uptake of VIG into everyday practice might be difficult, which is explored further in the following theme.

### Doing VIG work

This theme explores participants’ experiences of actually doing VIG work. Trainee Guiders appeared to struggle balancing carrying out VIG work and attending supervision sessions with the practical demands of their day-to-day roles. One senior manager felt that human resource support for VIG work should be managed at a local level:

*What we can’t have is a manager coming forward going “oh we didn’t release him [a trainee Guider]… because this popped up, or that popped up”, that needs to be managed separately* (Senior Manager 1)

There was a suggestion that at a local level, having dedicated ‘floating’ staff might help release trainee Guiders to be able to do their VIG work. However, the priority commanded by the perennial challenge of sickness cover within the organization meant that this suggestion might not be fruitful:

*a core of people who could cover for everything would be beneficial, but they would be sucked up before any VIG work was involved…we’ve had floating staff many times in the past, and they get sucked into long term sickness, and then that’s them gone* (Trainee Guider 2, teacher)

On a general level, the implementation of interventions within the organization at times seemed to be overwhelming for staff:

*we don’t let our staff get their breaths back from the first one [intervention] that we’ve just introduced before another one and another one and another one is introduced* (Trainee Guider 7, speech & language therapist)

This implementation strategy came to a head during the project in one area of the organization where another video-based intervention was introduced which focused directly upon service user behaviour, and made mandatory by senior management. There was a lack of co-ordination between the VIG project and the implementation of the other video intervention, which caused some confusion amongst staff. With regard to this other intervention, staff were:

*a bit daunted at first…because it’s another huge workload…but can see the benefits for the [service users]…so willing to put the effort in and go the extra mile* (Trainee Guider 2, teacher)

This quote suggested that sustained involvement of staff in this other intervention might have arisen partly as a result of a clear and immediate understanding of the expected benefits to the service users. This understanding also possibly helped staff to accept the mandatory imposition by management despite recognition that it would bring an increase in workload. In contrast, VIG was offered to staff as an optional intervention and offered to trainee Guiders as an optional training pathway and, as has already been shown, understanding of the expected benefits from VIG was less immediately clear. It therefore became more challenging to conduct VIG work in this area of the organization as it became somewhat marginalised by this other intervention which appeared to take priority.

These findings appeared to relate to the NPT construct of Collective Action (enacting the work required by the intervention). It seemed that there were at times severe barriers to trainee VIG Guiders being able to carry out the work required of them. These barriers seemed primarily to be concerned with organizational support, both at the local level for line managers, and also at a more strategic level around co-ordination of concurrent interventions. These barriers may be explored further in the next theme.

### Ideas for the future: new directions or the same path?

This theme explores participants’ thoughts about any potential future that VIG might have within the organization after the implementation project had officially ended. One trainee Guider who had completed training conveyed how VIG could lead to increased confidence in staff who then might develop more creative ideas for future practice, but questioned whether Organization A was receptive to ideas originating from ‘shop floor’ staff and thus whether these new ideas would be sustainable:

*a lot of our staff are bubbling with ideas but…they’re not given that network to really express their ideas or to see them go forward…VIG is really about that self-discovery, that, “oh, I can do this” and “what if we do that, what if we try this?”…to really see it blossom you have to be in that environment where those ideas are accepted and taken forward for it to truly be embedded* (Trainee Guider 7, speech & language therapist)

This concern that the organization may not be wholly receptive to creativity from its staff was highlighted by a quote from a senior managers who had not directly experienced VIG. This manager appeared to understand VIG as being an instructional training tool to demonstrate to all staff precisely how to work with a specific child:

*if you see it, how the staff present themselves, how they respond, how they interact, how they reciprocate interaction, how they use eye contact, tones of voice…[it is] very difficult to write that down but that’s how we want you to do it… if we can capture how that activity needs to be done in an autism-specific way to that child, then it’s universal* (Senior Manager 2)

This quote suggested that understanding VIG as a tool for reflective practice with emphasis upon enhancing personal relationships might have been challenging in an environment which favoured routine approaches ahead of supporting staff to be creative and trusting their own intersubjective skills.

In this theme, findings show that staff who had experienced and benefitted from VIG appeared to develop new thinking about future practice. However, a senior manager who had not been through VIG appeared to have a fundamentally different understanding of what it was. These findings highlighted the importance shown within the earlier themes of a reciprocal relationship between Coherence (understanding) and Cognitive Participation (involvement), and how this relationship might be further enhanced by Reflective Practice (evaluation). The senior manager was in a position of strategic influence within the organization, and thus it seemed possible that there might be a lack of organizational support (i.e. barriers to Collective Action) for novel ideas raised by staff which differed from current practice, perpetuating cultural resistance to change.

Table 4 summarises the ideal conditions for implementation of VIG according to the constructs of NPT, in comparison to the real conditions found in the study.

**Table 4:** Ideal and real conditions for implementation of VIG according to NPT constructs

NPT construct	Ideal conditions	Real conditions
Coherence: Making sense of the intervention	Able to differentiate the intervention from current practice; understanding the aims and expected benefits of the intervention	‘Positive’ focus of VIG not easy to understand, or believe, until directly involved. Confusion with other video intervention aimed at directly benefitting service users
Cognitive Participation: Becoming involved with the intervention	Key individuals driving the intervention forward, individuals’ belief that it is right and useful for them to be involved; keeping the intervention in view	Encouragement to receive VIG seen as better coming from staff who had experienced VIG, rather than from senior managers. Staff who had received VIG drove implementation by ‘selling’ the positive experience to colleagues, thus helping to dispel negative Coherence. Mixed picture about how much trainee Guiders incorporated VIG and its discursive principles into other practice
Collective Action: Enacting the work required by the intervention	Trainee Guiders able to have the time to carry out VIG work alongside daily role; management to support the co-ordination and organization of implementation	Trainee Guiders experienced problems in freeing themselves from daily roles in order to carry out VIG work. Proving staff to cover trainees likely to fail due to sickness cover priority. Senior manager believed logistical issues should be managed locally. Implementation of interventions more generally appeared to be overwhelming for staff with poor co-ordination
Reflexive Monitoring: Evaluating and appraising the intervention	Able to see the impact of the intervention; able to discuss the impact of the intervention; able to reshape practice as a result of the intervention	Staff who had received VIG directly experienced benefits (e.g. boost to confidence) which was visible to their colleagues. Suggestions of ideas for future practice shaped by influence of VIG, but speculation that these ideas might not come to fruition within perceived organizational culture

### What about the individual? The role of emotions within implementation

This theme explores some data which contained strong individual emotional responses to the implementation of VIG. It overlaps with earlier themes, which we may exemplify by re-examining two quotes presented earlier.

One quote has shown how one trainee Guider had been ill-prepared for her introduction to VIG Guider training:

*I got a phone call from my manager…saying I’d been put forward for VIG [Guider training]…I had to book my own place [on the induction course] and then I was given very little or no information until that day when I went up there… that was quite a horrendous experience… I was quite horrified because I felt like a fish out of water because I felt as if everybody knew what they were doing and I’m sitting there trying to catch up. And I’m quite a slow learner anyway, so I really, really, really felt out of my depth* (Trainee Guider 1, children’s home manager)

This trainee Guider was suggesting that better preparation would have helped her understanding and experience of getting involved. Via the theme ‘First steps: What is it all about?’, the quote was related to the NPT constructs of Coherence (understanding) and Cognitive Participation (involvement). However, on closer analysis the emotional content, tone of talk, and personal reflexivity did not seem to be captured fully within the NPT constructs, as they appeared to lean towards representation of the collective and were relatively under-specified with respect to the individual response or circumstance. The NPT constructs of Coherence and Cognitive Participation seemed less able to represent the personal, emotional processes through which individuals might arrive at their own understanding and involvement. So we also considered this quote with regard to the construct of Reflexive Monitoring (evaluation and appraisal), as this trainee Guider was reflecting upon her first experience of the intervention. However, here again, the construct of Reflexive Monitoring appeared to be more representative of the collective appraisals of the worth of the intervention for an organization, and appeared less helpful in capturing the personal mechanisms through which individuals make those appraisals.

Another quote questioned whether any impact of VIG would be sustained within the organization in the future:

*a lot of our staff are bubbling with ideas but…they’re not given that network to really express their ideas or to see them go forward…VIG is really about that self-discovery, that, “oh, I can do this” and “what if we do that, what if we try this?”…to really see it blossom you have to be in that environment where those ideas are accepted and taken forward for it to truly be embedded* (Trainee Guider 7, speech & language therapist)

This quote, via the theme ‘Ideas for the future: New directions or same path?’, seemed to correspond to all the constructs of NPT: for successful implementation, there was a need for Coherence (an understanding that VIG is about self-discovery); Cognitive Participation (there were many staff who are keen to be involved); Collective Action (there was a need for staff to be supported by the organization to experiment with new ideas); and Reflexive Monitoring (staff members’ enthusiasm arose from a confidence boost as a result of experiencing VIG). Yet the focus provided by the NPT constructs appeared to be about the collective, about the broader picture, with less insight into the mechanisms through which individuals might develop their own personal reflections. This quote could be considered to be this trainee Guider’s own Reflexive Monitoring; her evaluation of VIG and appraisal of the future potential within the organization after having been involved in the project. However, there appeared to be a sense of disappointment within her words, a perception that the future would see a lost opportunity because of perceived difficulties with the organizational culture. The construct of Reflexive Monitoring did not seem capable of capturing the personal mechanisms of how or why this trainee Guider arrived at such an evaluation.

## Discussion

This study explored the implementation of Video Interaction Guidance (VIG) within an organization delivering specialist autism care (Organization A) using Normalization Process Theory (NPT). The exploration was conducted from the perspectives of staff members of Organization A who were being trained to deliver the intervention, and senior managers influential in the implementation strategy of the intervention. We believe that it is one of the first studies to explore the implementation of VIG within a social care organization, with broader potential to enhance understanding of the implementation of similar psychosocial interventions. We also believe it is one of the first to use NPT with regard to the implementation of a complex intervention within care for people with autism. To our knowledge, prior to the present study, the only use of NPT in relation to autism care has been as an analytical framework for a scoping review of models of transitional care for young people with complex health needs [33].

Results suggest that VIG was believed to contribute towards empowering staff members, but that the implementation of VIG was challenging within the organizational culture. These two aspects, the impact of VIG and its implementation challenges, are discussed under ‘intervention strengths’ and ‘cultural fit’. Recommendations for future implementation of VIG, and reflections on use of NPT as a theoretical lens in this study, are also offered.

### Intervention strengths

The strongest finding was that participants reported that both their own and other staff members’ direct involvement in VIG allowed them to better understand VIG through experiencing its empowering benefits, which resulted in advocacy for the intervention within the organization. There appeared to be a change in mind-set from initial scepticism and wariness about VIG based on perception of the organizational culture as focusing upon negatives in practice, to recommending VIG as a positive, empowering experience. This new mind-set may be considered a form of peer advocacy in which the recommendation of VIG by a member of a staff team has credibility amongst their immediate colleagues in the team because that staff member shares the same ‘insider’ knowledge, experience and circumstances as fellow members of the team [34]. Recommendations of VIG from managers more removed from a staff team may seem less credible as they may not be granted this ‘insider’ status. Peer advocacy may thus be a potentially useful implementation strategy for VIG within an organization comparable to Organization A.

The mechanism underpinning this transformation in mind-set from sceptic to advocate may be located in the discursive, co-constructed pedagogy of VIG, which recognises individual people (in this case, the trainee Guiders and other staff members of Organization A) as experts in their own context. Such a style appeared to be empowering to both trainee Guiders learning how to deliver the intervention, and to staff receiving the intervention: As they found themselves given time and space to think and talk about their own emotions in relation to the video footage they were seeing, appeared to develop confidence in their abilities at their practice and develop more ideas for future practice. Development of skills in practising this discursive approach are at the fulcrum of VIG Guider training. The reports of staff members’ positive experiences of VIG; changes in attitudes from sceptics to advocates for VIG; and adoption of the discursive style honed in VIG Guider training into other areas of practice, all suggest that from an implementation perspective, VIG and its empowering ethos had the potential to become embedded within Organization A.

Despite this potential, conditions for embedding the intervention at a collective, organizational level did not appear to have been met. This short-fall appeared at least partly due to the speed at which change from VIG occurred. It seemed clear that changes occurred slowly, for two reasons: first, because the intervention itself was not a ‘quick fix’ and required time and investment; second, change occurred within individual staff members who had experienced VIG, i.e. one person at a time. This slow speed of change also seemed to have implications for the cultural fit of the intervention.

### Cultural fit

There were often implementation difficulties of a practical nature in securing the time and resources for participants to be able to carry out work required for their VIG Guider training. These difficulties appeared on the surface to arise from competing short-term priorities (e.g. sickness absence) and a lack of coordination in the general implementation of initiatives within the organization. It seemed possible that the slow speed of change of VIG highlighted above might have exacerbated these implementation difficulties, because VIG required a sustained practical resource commitment rather than short-term solutions. Another more underlying reason for these difficulties may have been a challenge of ‘fit’ between the ethos of VIG and the perceived organizational culture. This challenge of ‘fit’ was exemplified when one senior manager proffered strong ideas for the future of VIG at a strategic level, yet these ideas revealed an understanding of VIG as an instructional tool to enhance routine and consistency rather than recognising staff as experts in their own context and empowering them to shape their own practice. Creative ideas proposed by staff members empowered by VIG may have been stymied by an organizational culture which perhaps preferred to deliver instruction to staff about how to work, and which was not perceived to be wholly receptive to local ideas originating from ‘shop floor’ staff.

Theoretically, the concept of ‘structural empowerment’ within an organization (the transfer of authority and responsibility from management to staff) is recognised as a key predictor of individual, psychological empowerment [4]. One component of psychological empowerment may be known as ‘impact’ (i.e. an individual staff member’s perception of the degree to which he or she can influence outcomes at work) [35]. It seems straightforward to suggest that an organization with high levels of structural empowerment (i.e. an organization in which staff members are afforded influence in decisions) is likely to be an organization in which staff members feel that they can have high impact on outcomes at work. Organizational research suggests that staff members at all levels within an organization can differentiate between genuine organizational commitments to empowerment and management rhetoric about empowerment that is tokenistic [4]. There is perhaps a danger here with regard to VIG: empowering staff members of an organization which they themselves judge at times to offer disingenuous rhetoric about staff empowerment may result in these staff feeling dissatisfied and resentful at their lack of opportunity to explore their new-found confidence and ideas about their own practice [4]. A lack of advancement opportunities and feelings of unrecognition are cited as core reasons why staff members leave a workplace [36] and therefore it is also plausible to suggest that at the extreme, staff members empowered through VIG may become so frustrated with their perception of a misfit between their new outlook and the perceived organizational culture that they may ultimately seek to leave the organization to look for opportunities elsewhere. From an organizational point of view the latter scenario could be particularly damaging if the organization has invested financially in VIG as an intervention to empower staff, including the cost of training some of its staff to become VIG Guiders, only to find that these staff take their newfound skills and ideas elsewhere.

### Recommendations for implementation of VIG

One way to overcome the implementation challenges of VIG may be for those in positions of senior management to receive VIG directly themselves towards the beginning of an implementation project. Receiving VIG may allow the management to develop their understanding of the intervention, which would facilitate ‘radical, expansive exploration’ in their learning to create new knowledge and practice for the new and emerging activities required by the intervention [37]. Managers and leaders with high levels of emotional intelligence seem to be more effective, innovative and supportive of their teams [38]. If senior managers directly experience empowerment in their own practice as a result of receiving VIG, they may then be better equipped to support staff as they work with the intervention and as they attempt to explore new ways of working as a result of experiencing the intervention. This suggestion supports the recommendation made by Dowling et al. [5] for organizations to value all their staff (including managers) and provide them with tailored support.

### Reflections on the use of normalization process theory

This study reinforces the findings of others who have worked with NPT [39–42] regarding the importance of staff members’ understanding (‘Coherence’) and involvement (‘Cognitive Participation’) in embedding a complex intervention in practice, and challenges in establishing the conditions for successful implementation proposed by these two constructs. The initial analysis using inductive methods revealed the range of barriers and facilitators that were important to study participants in implementing VIG, and allowed for the emergence of key themes that are less central to the focus of NPT. As a higher level analysis however, NPT was used to develop a more sophisticated understanding of the processes of implementation at work in this context, by directing attention to some of the reciprocal relationships between the work that participants did (or needed to do), particularly with respect to understanding (Coherence) and involvement (Cognitive Participation). The study also reinforces the findings of others [40] in reconciling some data concerned with the emotional nature of professional work with the NPT framework. The NPT constructs appeared more attuned to collective processes regarding implementation, but appeared less attuned to the personal processes through which individuals made appraisals and reflections. This analysis suggests that NPT does not currently have enough specificity to explain how individual agency weaves with collective processes and structural systems to embed innovation in routine practice. Such a finding supports the suggestion by May [43] that enhancing understanding of implementation will require better understanding of the different insights which different theories offer to the implementation process, particularly the relationship between individual readiness for change and the actions individuals take to contribute to the change process. Despite some data appearing to be less closely aligned with the core focus of the NPT constructs, use of the theory has allowed some insight into implications of individual empowerment arising from VIG and the problematic fit of this individual empowerment within the organizational culture.

## Conclusions

This study has applied Normalization Process Theory (NPT) to explore the implementation of a constructivist, video-feedback intervention called Video Interaction Guidance (VIG) to empower staff with an autism care organization. Results suggest that VIG contributed towards empowering staff, but that the implementation of VIG and VIG Guider training was challenging within the organizational culture. The role of experience may be crucial to developing understanding of this type of intervention. Senior managers of the organization may need to experience the intervention to promote reflection in their own practice so that they are then able to provide staff with appropriate support during the implementation process. Further research is needed into interventions which aim to empower staff by incorporating a focus upon their emotional responses and resources, and how such interventions may fit within or potentially change the culture of the organization in which implementation occurs.

## Additional files

Additional file 1: Semi-structured interview prompts based on NPT constructs. (DOC 29 kb)
Additional file 2: Consolidated criteria for reporting qualitative studies (COREQ): 32-item checklist. (DOCX 17 kb)

## Abbreviations

NPT: Normalization process theory
VIG: Video interaction guidance

## References

[1] Francis R (2010). Independent Inquiry into Care Provided by Mid Staffordshire NHS Foundation Trust: January 2005 – March 2009.

[2] Francis R (2013). Report of the Mid Staffordshire NHS Foundation Trust Public Inquiry.

[3] Thorlby R, Smith J, Williams S, Dayan M (2014). The Francis Report: One Year On.

[4] Maynard MT, Gilson LL, Mathieu JE (2012). Empowerment – fad or fab? A multilevel review of the past two decades of research. J Manage.

[5] Dowling S, Manthorpe J, Cowley S (2006). Person-centred planning in social care: a scoping review.

[6] Rappaport J (1987). Terms of empowerment/Exemplars of prevention: Toward a theory for community psychology. Am J Commun Psychol.

[7] Dalrymple J, Boylan J (2013). Effective Advocacy in Social Work.

[8] Robertson J, Emerson E, Hatton C, Elliott J, McIntosh B, Swift P (2005). The Impact of Person Centred Planning.

[9] Butrimaviciute R, Grieve A (2014). Carers’ experiences of being exposed to challenging behaviour in services for autism spectrum disorders. Autism.

[10] Hellzen O, Asplund K (2002). Being in a fragmented and isolated world: interviews with carers working with a person with a severe autistic disorder. J Adv Nurs.

[11] Kennedy H, Landor M, Todd L (2011). Video Interaction Guidance: A Relationship-Based Intervention to Promote Attunement, Empathy and Wellbeing.

[12] Medical Research Council (2008). Developing and evaluating complex interventions: new guidance.

[13] Freire P (1970). Pedagogy of the Oppressed.

[14] Fukkink RG, Trienekens N, Kramer LJC (2010). Video feedback in education and training: putting learning in the picture. Educ Psychol Rev.

[15] National Institute for Health and Clinical Excellence. Social and emotional wellbeing: early years. Manchester: NICE public health guidance 40; 2012.

[16] National Institute for Health and Clinical Excellence. Autism: the management and support of children and young people on the autistic spectrum. Manchester: NICE clinical guidance 170; 2013.

[17] Hellier C. Adapting a model of delivery of Video Interaction Guidance to suit organizations beyond the normal boundaries of child/parent/educator. Educational Psychology in Scotland. 2014;15:59–63.

[18] Greenhalgh T, Robert G, MacFarlane F, Bate P, Kyriakidou O (2004). Diffusion of innovations in service organizations: systematic review and recommendations. Milbank Q.

[19] Health Foundation (2011). Evidence scan: improvement science.

[20] May C, Finch T (2009). Implementing, embedding, and integrating practices: An outline of Normalization Process Theory. Sociology.

[21] Murray E, Treweek S, Pope C, MacFarlane A, Ballini L, Dowrick C, Finch T (2010). Normalisation process theory: a framework for developing, evaluating and implementing complex interventions. BMC Med.

[22] May CR, Finch TL, Cornford J, Exley C, Gately C, Kirk S (2011). Integrating telecare for chronic disease management in the community: What needs to be done?. BMC Health Serv Res.

[23] Reeves S, Albert M, Kuper A, Hodges BD (2008). Why use theories in qualitative research?. Brit Med J.

[24] Eccles M (2006). The Improved Clinical Effectiveness through Behavioural Research Group. Designing theoretically-informed implementation interventions. Implement Sci.

[25] McEvoy R, Ballini L, Maltoni S, O’Donnell CA, Mair FS, MacFarlane A (2014). A qualitative systematic review of studies using the normalization process theory to research implementation processes. Implement Sci.

[26] James DM, Hall A, Phillipson J, McCrossan G, Falck C (2013). Creating a person-centred culture with the North East Autism Society: preliminary findings. Br J Learn Disabil.

[27] James DM, Hall A, Lombardo C, McGovern W (2015). A video feedback intervention for workforce development: exploring staff perspective using longitudinal qualitative methodology. J Appl Res Intellect.

[28] Association for Video Interaction Guidance UK (2015). Training to be a practitioner.

[29] Patton MQ (1990). Qualitative Evaluation and Research Methods (2^nd^ ed).

[30] Braun V, Clarke V (2006). Using thematic analysis in psychology. Qual Res Psychol.

[31] MacFarlane A, O’Reilly-de BM (2012). Using a Theory-Driven Conceptual Framework in Qualitative Health Research. Qual Health Res.

[32] Lincoln YS, Guba EG (1985). Naturalistic Inquiry.

[33] Watson R, Parr JR, Joyce C, May C, Le Couteur AS (2011). Models of transitional care for young people with complex health needs: A scoping review. Child Care Hlth Dev.

[34] Atkinson D (1999). Advocacy: a review.

[35] Spreitzer GM (1995). Psychological empowerment in the workplace: dimensions, measurement and validation. Acad Manage J.

[36] Branham L (2012). The seven hidden reasons employees leave: how to recognize the subtle signs and act before it’s too late.

[37] Engeström Y (2004). New forms of learning in co-configuration work. J Workplace Learning.

[38] Erkutlu H, Chafra J (2012). The moderating roles of leader’s emotional intelligence and proactive personality. Health Organ. Manag..

[39] Gunn JM, Palmer VJ, Dowrick CF, Herrman HE, Griffiths FE, Kokanovic R (2010). Embedding effective depression care: using theory for primary care organizational and systems change. Implement. Sci.

[40] Gallacher K, May CR, Montori VM, Mair FS (2011). Understanding Patients' Experiences of Treatment Burden in Chronic Heart Failure Using Normalization Process Theory. Ann Fam Med.

[41] Sanders T, Foster NE, Ong BN (2011). Perceptions of general practitioners towards the use of a new system for treating back pain: a qualitative interview study. BMC Med.

[42] Franx G, Oud M, de Lange J, Wensing M, Grol R (2012). Implementing a stepped-care approach in primary care: results of a qualitative study. Implement Sci.

[43] May C (2013). Towards a general theory of implementation. Implement Sci.

[44] Murray L, Trevarthen C, Field TM, Fox NA (1985). Emotional regulation of interactions between two-month-olds and their mothers. Social perception in infants.

[45] Trevarthen C, Aitken KJ (2001). Infant intersubjectivity: research, theory and clinical applications. J Child Psychol Psych.

[46] Vygotsky LS, Cole M, John-Steiner V, Scribner S, Souberman E (1978). Interaction between learning and development. Mind in society: The development of higher psychological processes.

[47] Wood D, Bruner JS, Ross G (1976). The role of tutoring in problem solving. J Child Psychol Psych.

