# Supplementary material for: Implementing a video-based intervention to empower staff members in an autism care organization: a qualitative study
Source: BMC Health Serv Res. 2016 Oct 21;16:608. doi: 10.1186/s12913-016-1820-9 (PMC5073918; doi:10.1186/s12913-016-1820-9)
Supplement: Additional file 1: — Semi-structured interview prompts based on NPT constructs. (DOC 29 kb) [file 12913_2016_1820_MOESM1_ESM.doc]

**Additional file 1:** Semi-structured interview prompts based on NPT constructs

| **NPT Construct** | **Questions/Prompts** |
| --- | --- |
| **Coherence:** making sense of the intervention | What was your understanding of VIG when you were introduced to it?  I’d like you to think back to when you first heard about VIG, what did you think it was?  What role did you see for VIG within [name of organization]?  How did you see VIG fitting into [name of organization] given the other interventions and development opportunities that exist?  What benefits did you think that VIG would bring to [name of organization]?  Who did you think would benefit from VIG? |
| **Cognitive Participation:** involvement with the intervention | Whose involvement do you see as necessary for VIG to have maximum impact?  What do you see your role as being?  Describe the people who you have come into contact with and worked with since you began your VIG training? |
| **Collective Action:** how practical work of *doing* the intervention is carried out within the organization | Tell me how you find the VIG work?  Can you tell me how a piece of VIG work gets done, from start to finish?  Do you feel VIG is compatible with existing practice?  Do you think that VIG fits with the goals and activity of the organization? |
| **Reflexive Monitoring:** evaluation and appraisal of the intervention | Now that you have been directly involved with VIG what role do you see for VIG?  Have your thoughts about VIG changed now that you have been directly involved?  Thinking about doing your VIG work, is there anything that would this easier for you?  Is there one thing/anything you would change about VIG training?  Do you think VIG has had any impact within the organization? |
